# Prostate cancer with synchronous metastatic penile lesion: A case report

**DOI:** 10.1016/j.eucr.2025.103172

**Published:** 2025-08-19

**Authors:** Muhammad Fajar, Ikhlas Arief Bramono, Fakhri Rahman, Farilaila Rayhani, Edward Usfie Harahap, Rachmat Budi Santoso

**Affiliations:** a Department of Urology, Faculty of Medicine Universitas Indonesia, Cipto Mangunkusumo Hospital, Jakarta, Indonesia; b Department of Urology, National Cancer Center Dharmais Cancer Hospital, Jakarta, Indonesia; c Department of Anatomical Pathology, National Cancer Center Dharmais Cancer Hospital, Jakarta, Indonesia

**Keywords:** Bone metastasis, Penile lesion, Prostate cancer, Metastasis-directed therapy

## Abstract

Prostate cancer with penile metastasis is exceedingly rare. We report a synchronous metastatic hormone-sensitive prostate cancer presenting initially as a penile lesion in a 70-year-old male. Penile tumor excision confirmed prostate adenocarcinoma by immunohistochemistry. MRI revealed prostate enlargement without lymph node involvement; biopsy indicated Gleason Score 3 + 3 adenocarcinoma, and bone scan showed pubic bone metastasis. Initial PSA was significantly elevated (318 ng/ml). Androgen deprivation therapy led to progressive PSA decline and good clinical response. Aggressive surgical intervention for penile metastasis is discouraged due to limited benefits and potential deterioration in quality of life.

## 1 Introduction

Prostate cancer is the second most common malignancy in men, affecting 1 in 8 males globally and strongly age-related, with a prevalence rising from 5 % (<30 years old) to 59 % (>79 years old). Although early stages are often asymptomatic, advanced stages can present with urinary retention and back pain.^1^, ^2^, ^3^

A 2022 study reported that approximately half of prostate cancer patients developed distant metastases, with bone being the most common site, followed by lymph nodes, liver, and thorax.^3^^,^^4^ Rare metastatic sites include the retroperitoneum, digestive system, kidneys, brain, and penis.^5^ Penile metastasis is extremely rare (0.3–0.5 %) and often misdiagnosed due to nonspecific symptoms such as nodules, ulceration, urinary complaints, or priapism.^5^^,^^6^ We present a case of synchronous metastatic hormone-sensitive prostate cancer presenting with a penile lesion as the initial symptom.

## 2 Case presentation

A 70-year-old man presented with a penile mass that had developed over the past year. Two accompanying symptoms developed over time: painful urination and difficulty urinating. The patient had previously undergone penile tumor surgery, with histopathology suggesting adeno-squamous carcinoma. History of heart disease, diabetes, and other medical conditions was denied.

**Fig. 1:**
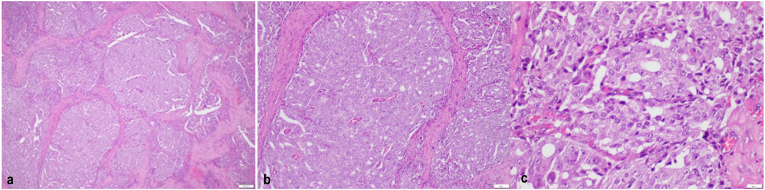
Histopathology with Hematoxylin-Eosin staining (A) 4× magnification (B) 10× magnification (C) 40× magnification. The tumor shows round to oval tumor cells arranged in groups forming cribriform and glandular. Pleomorphic nuclei, hyperchromatic, partially with nucleoli, and mitosis can be found.

**Fig. 2:**
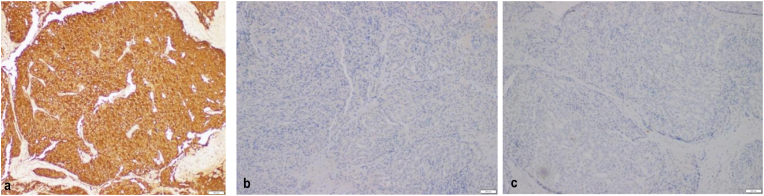
Immunohistochemistry Assay. (A) PSA immune reactivity, (B) no expression of CK7, and (C) no expression of CK20.

**Fig. 3:**
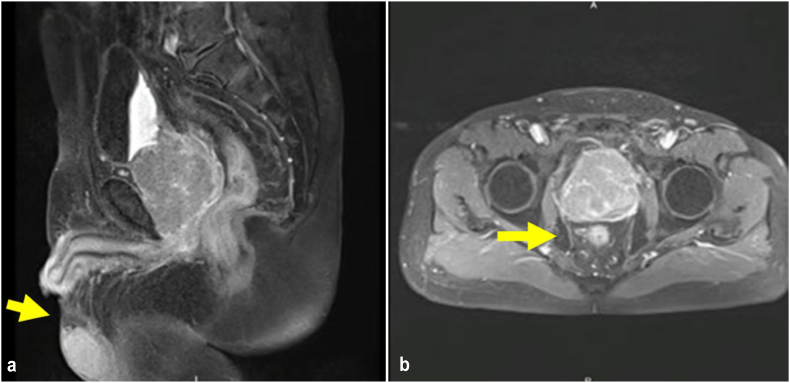
(A) Granulation tissue on the shaft penis; (B) Prostate enlargement.

Physical examination revealed a fixed palpable mass with a hard consistency on the penile shaft (granulation tissue post penile tumour excision). Following the examination, the previous histopathological result was re-examined in our hospital to confirm its origin. The findings showed different result with where the pathologists suggest an adenocarcinoma without lympho-vascular invasion (Fig. 1). Immunohistochemistry assay showed PSA immunereactivity, no expression of CK7, and no expression of CK20 **(**Fig. 2**)**. MRI revealed a 1.4 × 1 cm lesion on the left penis and prostate hypertrophy (7.5 × 6.1 × 7.3 cm). No recurrent lesions, intra-abdominal abnormalities, and lymphadenopathies were found (Fig. 3) (see Fig. 2).

Prostate specific antigen (PSA) level was 318 ng/ml. A biopsy of prostate tissue showed the features of prostate tissue accompanied by tumour mass, mitotic figures, infiltrative glands, and eosinophilic cytoplasm. Desmoplastic stroma with vessel dilatation was also found. However, no lymph or vascular invasion were identified. It was concluded that the result was adenocarcinoma of the prostate with minimal Gleason score 3 + 3 = grade group 1.

**Fig. 4:**
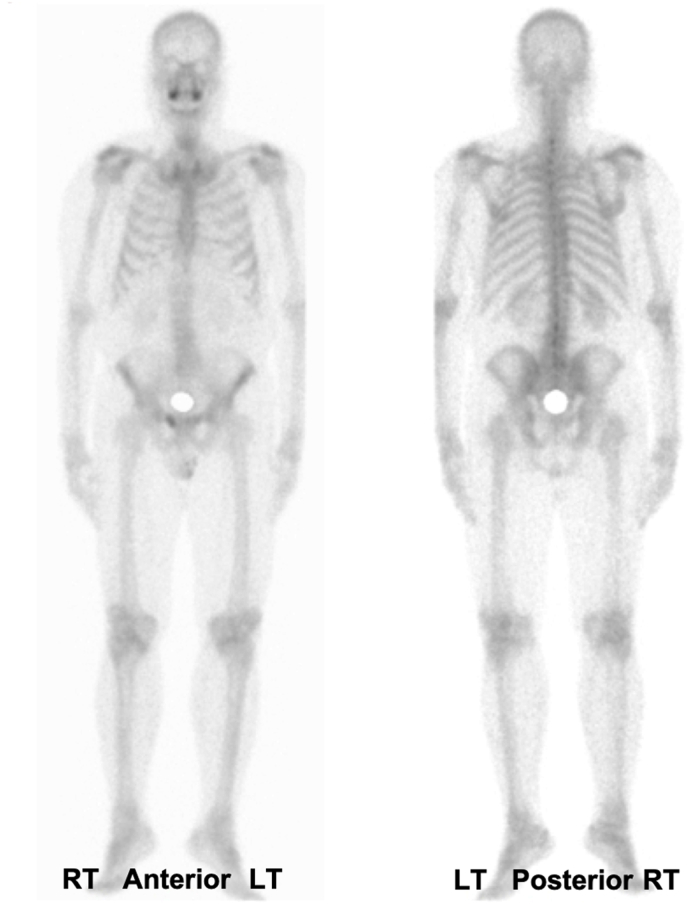
Full body bone scan.

A static whole-body bone scan was conducted, where pathological uptake of radioactivity is evident in the superior ramus of the right pubic bone was revealed. In contrast, the uptake of radioactivity in other bones appears to be relatively symmetrical and uniform. These findings support the impression and suggest the possibility of metastasis to the right pubic bone (Fig. 4).

The patient was diagnosed as metastatic prostate cancer. He was started on androgen deprivation therapy (ADT) and routinely attends his scheduled hospital visits for evaluation of the treatment response. PSA levels decreased to 93.16 ng/ml at 3 months and 18.91 ng/ml at 6 months post-ADT. Since this is a low metastatic burden prostate cancer case, with no sign of residual mass in the penis and single lesion on the pubic bone, we metastasis-directed therapy (MDT) with radiation to the prostate and bone lesion.

## 3 Discussion

Prostate cancer is the second most common malignancy in the male population.^1^^,^^7^ According to data from the American Cancer Society, an estimated proportion of 1 in 8 men will experience a prostate cancer diagnosis within their lifespan.^8^ The incidence rate increases up to 1 in every 52 men for ages 50–59 years. The incidence rate is nearly 60 % in men over the age of 65 years.^1^

Metastasis of prostate cancer to the penis is uncommon, with an incidence between 0.3 % and 0.5 %.^5^ Thus far, less than 500 cases have been reported in the literature, with the first described by Eberth in 1870.^9^ A study reported that the most frequent sites of metastases were bone (32.5 %), lung (9.6 %), lymph nodes (7.05 %), and liver (4.75 %).^4^ Our case's primary manifestation was penile metastasis, with radiological confirmation of additional metastasis in the right pubic bone.

Bone metastasis commonly occurs, particularly in the axial skeleton including ribs, pelvis, and spine due to red marrow abundance.^10^ The presence of bone metastasis indicates advanced disease, carrying prognostic and therapeutic implications.^11^ Paquin and Roland were the first to describe the potential routes of metastasis to the penis, including retrograde venous and lymphatic routes, arterial spread, direct extension, and surgical implantation.^12^

Synchronous penile and bone metastasis is rare.^7^An autopsy found 90 % of men with metastatic prostate cancer had bone involvement.^9^Common penile sites include shaft, root, and glans^7^. Penile metastases typically occur in the shaft and root.^7^ Treatment varies based on tumor extent and patient condition and includes local excision, penectomy, and chemotherapy. However, penile metastases usually signify disseminated disease with poor prognosis. Median survival is 9 months, with rare cases reaching 62 months.^13^

Liu et al. reported that patients with bone metastasis had 3- and 5-year survival rates of 47.7 % and 32.4 %, respectively, while those without had 98.4 % and 97.3 %.^11^

Primary treatments include surgery and radiotherapy, which may impact quality of life through erectile dysfunction, ejaculation changes, or incontinence.^14^, ^15^, ^16^

Radiation therapy is often combined with ADT. Loppenberg et al. found that patients receiving local therapy had a 3-year mortality-free survival of 69 % versus 54 % without.^17^ Other studies also highlight survival benefits from local therapy.^18^^,^^19^

MDT is gaining interest for oligometastatic prostate cancer (≤3 metastases), utilizing surgery, ablation, or SABR.^20^

Our case showed promising results with ADT. Simultaneous follow-up of PSA levels showed gradually decreasing PSA levels. The patient also did not report any new symptoms. Since this is a palliative case (stage 4 prostate cancer), the treatment provided so far was aimed at life prolongation rather than aggressive intervention.

## 4 Conclusion

Penile metastasis from prostate cancers is an extremely rare condition. Patients have a poor prognosis due to the low survival rate. Aggressive surgical treatment is not recommended as they are unlikely to provide substantial benefits and may further deteriorate quality of life.

## CRediT authorship contribution statement

**Muhammad Fajar:** Writing – review & editing, Writing – original draft, Visualization, Validation, Supervision, Software, Resources, Project administration, Methodology, Investigation, Funding acquisition, Formal analysis, Data curation, Conceptualization. **Ikhlas Arief Bramono:** Writing – review & editing, Writing – original draft, Visualization, Validation, Supervision, Software, Resources, Project administration, Methodology, Investigation, Funding acquisition, Formal analysis, Data curation, Conceptualization. **Fakhri Rahman:** Writing – review & editing, Writing – original draft, Visualization, Validation, Supervision, Software, Resources, Project administration, Methodology, Investigation, Funding acquisition, Formal analysis, Data curation, Conceptualization. **Farilaila Rayhani:** Writing – review & editing, Writing – original draft, Visualization, Validation, Supervision, Software, Resources, Project administration, Methodology, Investigation, Funding acquisition, Formal analysis, Data curation, Conceptualization. **Edward Usfie Harahap:** Writing – review & editing, Writing – original draft, Visualization, Validation, Supervision, Software, Resources, Project administration, Methodology, Investigation, Funding acquisition, Formal analysis, Data curation, Conceptualization. **Rachmat Budi Santoso:** Writing – review & editing, Writing – original draft, Visualization, Validation, Supervision, Software, Resources, Project administration, Methodology, Investigation, Funding acquisition, Formal analysis, Data curation, Conceptualization.

## Funding sources

His research did not receive any specific grant from funding agencies in the public, commercial, or not-for-profit sectors.
